# Discrepancies between physician-assessed and patient-reported complications after cystectomy – a prospective analysis

**DOI:** 10.1007/s00345-025-05487-7

**Published:** 2025-02-10

**Authors:** Benedikt Ebner, Judith Hirsch, Annkathrin Holz, Yannic Volz, Lennert Eismann, Julian Hermans, Nikolaos Pyrgidis, Marc Kidess, Marie Semmler, Isabel Brinkmann, Can Aydogdu, Michael Chaloupka, Andrea Katharina Lindner, Philipp Weinhold, Christian G. Stief, Gerald B. Schulz

**Affiliations:** Department of Urology, University Hospital, Ludwig-Maximilians-University Munich, Munich, Germany

**Keywords:** Cystectomy, Complications, Clavien-Dindo classification, Comprehensive Complication Index, Patient reported outcome, Optimist, Pessimist, Preoperative medical information

## Abstract

**Purpose:**

Despite the high incidence of perioperative complications following cystectomy, there is a lack of evidence regarding patients’ perceptions. Moreover, discrepancies between established complication grading systems and the patient’s perspective remain unexplored.

**Methods:**

We prospectively evaluated perioperative complications after cystectomy using the Clavien-Dindo Classification (CDC) and the Comprehensive Complication Index (CCI). The CDC and CCI were compared to patient-reported complication grades through Spearman correlation analysis. Discrepancies between physician-assessed and patient-reported complication grades were then evaluated. The study was registered at ClinicalTrials.gov (NCT05153694).

**Results:**

Between December 2021 and March 2024, 172 patients underwent open cystectomy at our department. Of those, 154 provided written consent to participate in the study, and 111 completed the post-discharge questionnaire. We found a moderate correlation between physician-assessed and patient-reported complication grades (CDC: *r* = 0.34, CCI: *r* = 0.39; *p* < 0.001). Patients with matching grades were defined as realists (50%). Those who reported lower complication grades than assessed by physicians were defined as optimists (38%), while those who reported higher grades were defined as pessimists (12%). Optimists rated the preoperative medical information better than pessimists (“very good”: 79% vs. 38%, *p* = 0.006). We found no significant differences between optimists and pessimists regarding age, gender, tumor characteristics or educational level.

**Conclusion:**

In our prospective study, the correlation between physician-assessed and patient-reported complication grades was only moderate. Only half of the cystectomy patients accurately perceived the severity of their complications. Our findings represent the first study to investigate patients’ perspectives on complications in urology and underscore the importance of thorough preoperative medical information.

**Supplementary Information:**

The online version contains supplementary material available at 10.1007/s00345-025-05487-7.

## Introduction

A cystectomy is associated with a high incidence of postoperative complications [1]. The Clavien-Dindo Classification (CDC) and the Comprehensive Complication Index (CCI) enable surgeons to grade these events [2, 3]. Both grading systems have been validated in urologic surgery [4–6]. The CDC is the most widely used complication reporting system in surgery and classifies complications into five grades with increasing severity, based on the invasiveness of the required treatment [6]. In a meta-analysis of 44 studies of patients undergoing radical cystectomy (RC), 62% of patients had some kind of complication according to the CDC [1]. The CCI is based on the CDC and describes the cumulative burden of postoperative complications with a continuous overall score from 0 (no complications) to 100 (death). In an analysis of 172 RC patients, the mean CCI was 26.3 [5]. Despite these high complication rates, there is still no existing evidence about the patient´s perspective on the severity of complications following cystectomy.

Studies in other surgical fields, like spine and hernia surgery, have highlighted a significant discrepancy between patient-reported outcomes and traditional physician-assessed grading systems [7–11]. Rendell et al. conducted a survey among 168 patients undergoing general surgery asking for the patient´s perspective on hypothetical postoperative complications [11]. Patients rated some minor complications as more severe than some major complications [11]. For instance, 37% of patients considered a blood transfusion (Clavien-Dindo grade 2) to be ‘a great deal’, which is significantly higher than the 8.9% who felt the same about a complication requiring endoscopy (Clavien-Dindo grade 3) [11].

To foster a trustful doctor-patient relationship and reduce dissatisfaction due to complications, understanding the patient’s perspective is crucial. We present the first study assessing this perspective on postoperative complications in urology. We prospectively compared physician-assessed and patient-reported grading of in-hospital complications following cystectomy and evaluated the impact of age, gender, tumor characteristics, educational level and preoperative medical information on discrepancies.

## Patients and methods

### Study design and patient cohort

**Fig. 1:**
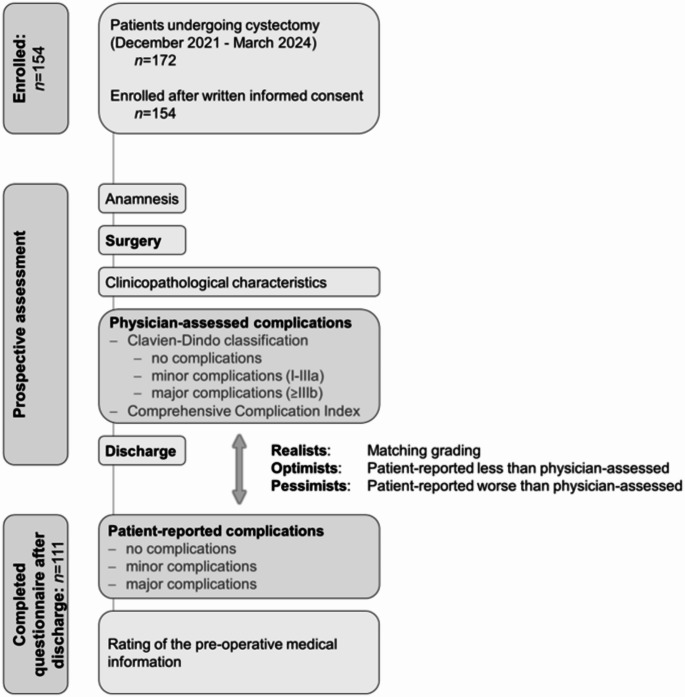
Flowchart of the prospective study design. We prospectively evaluated perioperative complications in cystectomy patients using the Clavien-Dindo classification and the Comprehensive Complication Index. After discharge, we asked all patients for a subjective assessment of in-hospital complications. Patients with matching complication grades were defined as realists. Those who reported lower complication grades than assessed by physicians were defined as optimists, while those who reported higher grades were defined as pessimists

We prospectively included patients undergoing open cystectomy for oncological and non-oncological indications from December 2021 to March 2024 at LMU University Hospital Munich. Written informed consent was obtained from all patients before enrollment. Clinicopathological and sociodemographic data were collected during the hospital stay. The study design is detailed in Fig. 1.

Perioperative care followed standardized protocols based on Enhanced Recovery After Surgery (ERAS) recommendations. No routine preoperative bowel preparation was performed. The nasogastric tube was removed immediately post-surgery. Patient-controlled epidural analgesia was administered routinely, and prokinetic agents were given from the first postoperative day. A neocystogram or conduitogram was performed after 10–14 days before removing the ureteric stents and catheter.

Regarding the grading of complications, we adhered to the EAU guidelines on muscle-invasive and metastatic bladder cancer [12]. In-hospital complications were graded using the Clavien-Dindo classification, and the Comprehensive Complication Index (CCI) was calculated (cci-calculator.com). To capture patients’ perspectives, telephone interviews with standardized questions were conducted three months post-discharge (Supplementary Table 1). Patients categorized their hospital stay as ‘no complications,’ ‘minor complications,’ or ‘major complications.’ Only in-hospital complications of the immediate postoperative period were evaluated, and patients were explicitly told that this was the sole focus. We included patients who responded to our calls and excluded those who did not respond after five attempts.

Patients with concordant physician-assessed (CDC) and patient-reported complication grades were categorized as realists, those reporting lower grades as optimists, and those reporting higher grades as pessimists. The study complied with the Declaration of Helsinki, was approved by the university ethics committee (project number: 18–059), and was registered on clinicaltrials.gov (NCT05153694).

### Outcomes

The primary outcome of this study was to determine the correlation between physician-assessed and patient-reported complication grades in patients undergoing cystectomy. Secondary outcomes included (i) the influence of clinicopathological characteristics on the discrepancies between physician-assessed and patient-reported complication grades, and (ii) the effect of preoperative medical information on these discrepancies.

### Statistical analysis

Continuous variables were shown as median with interquartile range (IQR) and categorical variables were shown as proportions. A two tailed t-test for independent samples was performed for comparisons between continuous variables. Before every t-test, a Levene test was performed to confirm variance equality. Chi-square tests were used to assess the association between categorical variables. If the assumptions of the chi² test were not met (e.g., cell frequencies less than 5), a Fisher’s exact test was performed. The significance level was set at 5%. All statistical analyses were conducted with DATAtab Team (DATAtab e.U. Graz, Austria).

## Results

### Clinicopathological and sociodemographic characteristics

Between December 2021 and March 2024, 172 patients underwent cystectomy at our department. Of these, 154 gave their written consent to participate in the study and were enrolled. After discharge, 111 patients took part in the telephone interview and were included in the analysis. Clinicopathological and sociodemographic characteristics are shown in Table 1. Overall, the median patient age was 71.3 years, and 85% of patients were male. The median BMI was 25.7 kg/m². According to the physical status classification system of the American Society of Anesthesiologists (ASA), 76% of patients had a severe systemic disease (ASA class > 2). Most patients (89%) had an oncological indication for cystectomy. Non-malignant indications for cystectomy included 7 patients (4.4%) with therapy-resistant hemorrhagic/radiation cystitis, 6 patients (3.8%) with therapy-resistant urethral strictures, and 3 patients (1.9%) with therapy-resistant bladder fistula formation. The histopathological examination showed organ-confined tumor growth (< T3a) in 66%, positive lymph nodes in 19% and a positive surgical margin in 9.4% of cases. The median operation time was 223 min. Blood transfusions were administered in 29% of patients. Of all patients, 45% had a tertiary education (International Standard Classification of Education level > 4). The pre-operatively provided medical information was rated very good in 66%, good in 22%, satisfactory in 8.3%, sufficient in 1.8%, and insufficient in 1.8% of cases.

**Table 1:** Overall clinicopathological and sociodemographic characteristics of patients undergoing cystectomy. Values are presented as median ± interquartile range or *n* (%). Abbreviations: BMI: body mass index. ASA: American Society of Anesthesiologists. IMCU: intermediate care unit. ICU: intensive care unit. ISCED: International Standard Classification of Education. Neoadjuvant chemotherapy: patients with urothelial cancer considered

Characteristic	Overall, *n* = 111 (100%)
Age (years)	71.3 (± 14.4)
Gender	
Male	94 (85%)
Female	17 (15%)
BMI (kg/m²)	25.7 (± 5.5)
ASA class > 2	84 (76%)
Oncological indication	99 (89%)
Neoadjuvant chemotherapy	17/97 (18%)
Urinary diversion	
Ileal conduit	74 (67%)
Ileal neobladder	36 (32%)
Ureterocutaneostomy	1 (0.9%)
Organ-confined tumor (< T3a)	65/97 (67%)
Positive lymph nodes	17/89 (19%)
Positive surgical margin	9/96 (9.4%)
Operation time (min)	223 (± 97.5)
Blood transfusion	32 (29%)
IMCU/ICU nights	1 (± 1)
Hospital stay (days)	18 (± 6.5)
Educational level (ISCED) > 4	50 (45%)
“Very good” preoperative medical information	72 (66%)

### Physician-assessed grading of complications

**Fig. 2:**
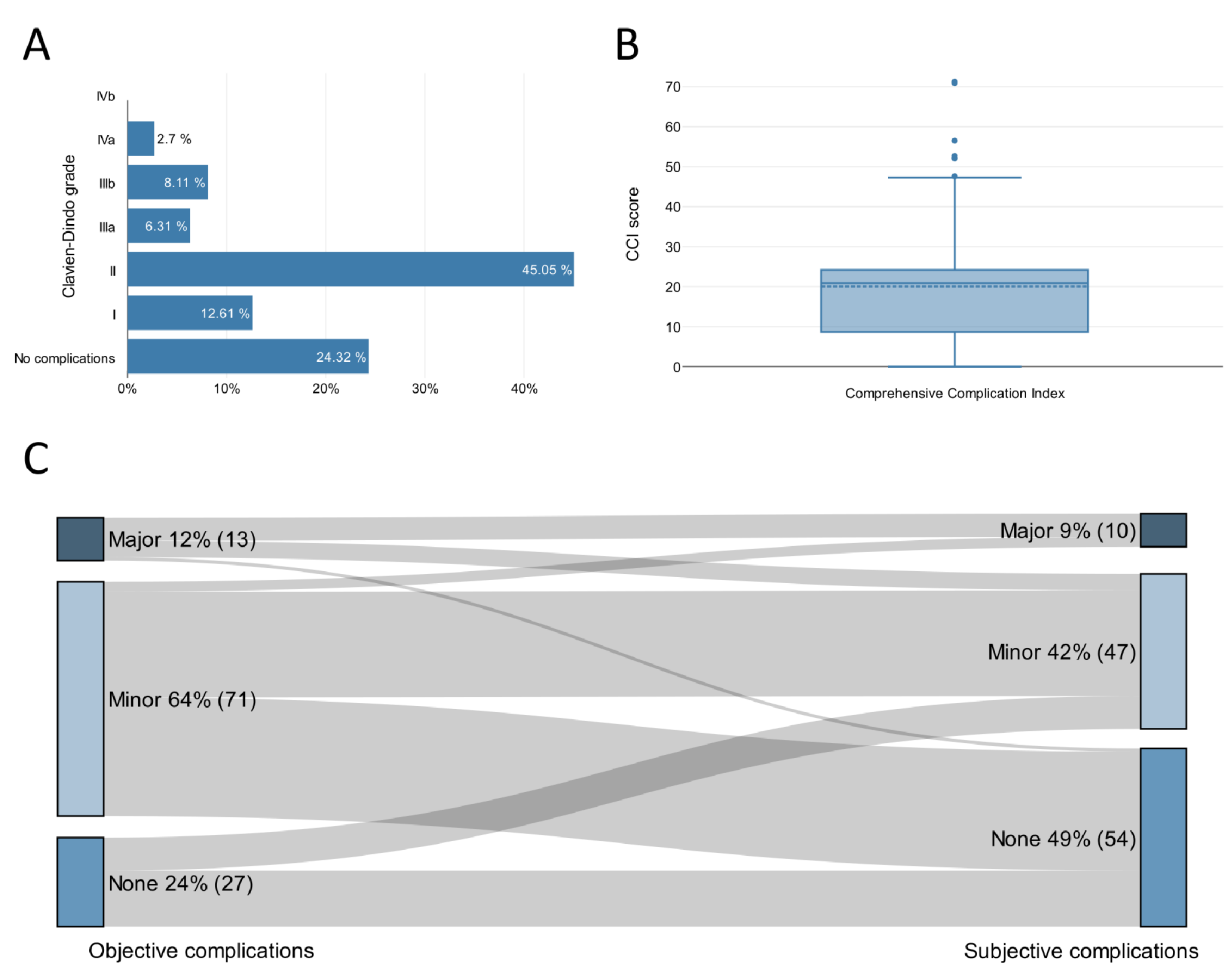
Highest Clavien-Dindo Classification **(A)** and Comprehensive Complication Index **(B)** of patients undergoing cystectomy. Sankey diagram of physician-assessed vs. patient-reported complications **(C)**: For the physician-assessed grading, the Clavien-Dindo system was used (minor complication: grade I-IIIa, major complication: grade ≥IIIb). After discharge, patients were asked if they subjectively experienced no, minor or major complications. Abbreviations: CDC: Clavien-Dindo Classification. CCI: Comprehensive Complication Index

We observed a total of 197 complications in 84 of 111 patients (76%). According to the CDC, 24% of patients had no complications, 64% had minor complications (CDC grade I-IIIa) and 12% had major complications (CDC grade ≥ IIIb) (Fig. 2A). The median CCI was 20.9 (IQR 15.5) (Fig. 2B). There was a very high correlation between CDC and CCI in the Spearman correlation analysis (*r* = 0.95, *p* < 0.001).

The most common CDC complications for each grade were fever (CDC grade I, 41% of patients), additional antibiotic therapy on top of the hospital standard (CDC grade II, 59% of patients), CT guided percutaneous drainage (CDC grade IIIa, 9.0% of patients), re-laparotomy (CDC grade IIIb, 3.6% of patients) and cardiac/respiratory failure requiring ICU management (CDC grade IVa, 2.7% of patients). We did not observe a CDC grade IVb complication (multi-organ dysfunction requiring ICU management, but not leading to death). Of all initially enrolled patients (*n* = 154), four patients (2.6%) died during the hospital stay (CDC grade V). However, due to the design of the study (patient-reported assessment of complications after discharge), there were no CDC grade V patients in the final analysis.

### Patient-reported grading of complications

Subjectively, 49% of patients reported no complications, 42% reported minor and 9% reported major complications (Fig. 2C). Both CDC and CCI only showed a moderate correlation with the patient-reported grading of complications in the Spearman correlation analysis (CDC: *r* = 0.34, *p* < 0.001; CCI: *r* = 0.39, *p* < 0.001).

### Realists, optimists and pessimists

In our study, 50% of patients were realists, 38% optimists and 12% pessimists (Fig. 2C). The comparison of clinicopathological and sociodemographic characteristics of optimists and pessimists is shown in Table 2. Optimists had a significantly higher body mass index than pessimists (27.3 ± 4.0 vs. 24.0 ± 3.6, *p* = 0.012). Preoperative information was rated significantly more often as “very good” by optimists compared to pessimists (79% vs. 38%, *p* = 0.006). There were no significant differences regarding age, gender distribution, tumor characteristics, length of the hospital stay or educational level between optimists and pessimists.

**Table 2:** Clinicopathological and sociodemographic characteristics of realists, optimists and pessimists. Patients who reported lower complication grades than assessed by physicians were defined as optimists, while those who reported higher grades were defined as pessimists. Patients with matching grades were defined as realists. Values are presented as median ± interquartile range or *n* (%). Abbreviations: BMI: body mass index. ASA: American Society of Anesthesiologists. IMCU: intermediate care unit. ICU: intensive care unit. ISCED: International Standard Classification of Education. Neoadjuvant chemotherapy: patients with urothelial cancer considered. The *p*-values shown refer to the comparison between optimists and pessimists

Characteristic	Realists, *n* = 56 (50%)	Optimists, *n* = 42 (38%)	Pessimists, *n* = 13 (12%)	*p*-value
Age (years)	70.2 (± 15.9)	70.6 (± 10.6)	75.9 (± 10.9)	0.52
Gender				
Male	45 (80%)	38 (90%)	11 (85%)	0.62
Female	11 (20%)	4 (10%)	2 (15%)	
BMI (kg/m²)	25.6 (± 5.4)	26.5 (± 4.6)	24.2 (± 3.8)	**0.012**
ASA class > 2	43 (77%)	30 (71%)	11 (85%)	0.48
Oncological indication	50 (89%)	36 (86%)	13 (100%)	0.32
Neoadjuvant chemotherapy	8/49 (16%)	7/35 (20%)	2/13 (15%)	0.72
Urinary diversion				
Ileal conduit	39 (70%)	29 (69%)	6 (46%)	0.07
Ileal neobladder	17 (30%)	13 (31%)	6 (46%)	
Ureterocutaneostomy	0 (0%)	0 (0%)	1 (7.7%)	
Organ-confined tumor (< T3a)	30/50 (60%)	25/36 (69%)	10/13 (77%)	0.81
Positive lymph nodes	9/47 (20%)	6/30 (20%)	2/12 (17%)	1.00
Positive surgical margin	6/50 (12%)	2/36 (5.6%)	1/13 (7.7%)	1.00
Operation time (min)	211.5 (± 110.8)	229 (± 94)	207 (± 79)	0.36
Blood transfusion	17 (30%)	10 (24%)	2 (15%)	0.71
IMCU/ICU nights	1 (± 1)	1 (± 1)	0 (± 1)	0.18
Hospital stay (days)	18 (± 6)	17.5 (± 6)	18 (± 4)	0.67
Educational level (ISCED) > 4	26 (46%)	18 (43%)	6 (46%)	1.00
“Very good” preoperative medical information	32 (57%)	33 (79%)	5 (38%)	**0.006**

## Discussion

### Physician-assessed grading of complications

In our study, we prospectively assessed postoperative complications with two different grading systems that have both been validated in urological surgery [4–6]. The CDC is the most widely used complication reporting system in surgery [6]. In our study, 64% of patients had minor complications (CDC grade I-IIIa) and 11% had major complications (CDC grade IIIb-V). These findings are consistent with those reported in a systematic review and meta-analysis of 44 studies on complications following RC [1]. The review reported that 52% of patients had minor complications (CDC grade I-IIIa) and 10% had major complications (CDC grade IIIb-V) [1]. The median CCI in our study was 20.9 (IQR 15.5). This was also in the same range as previously reported in a CCI validation study of 172 patients undergoing radical cystectomy (mean CCI: 26.3 ± 20.8 SD) [5].

### Correlation of physician-assessed and patient-reported grading of complications

There was a very high correlation between the CDC and the CCI in the Spearman correlation analysis, but both physician-assessed systems only showed a moderate correlation with the patient’s perspective. It has been shown before in other surgical fields like spine and hernia surgery, that patient-rated complication severity significantly differs from objective grading systems [7–11]. There are no studies on this topic in urology to date. In our study, there was a discrepancy between physician-assessed and patient-reported complication grades in every second case. Overall, complications were better tolerated than expected, as half of the patients reported no complications at all (Fig. 2C).

### Optimists, realists and pessimists

Every second patient had a realistic perception of the severity of postoperative complications (match between physician-assessed (CDC) and patient-reported complication grade). The number of optimists was three times higher than the number of pessimists. Surprisingly, we did not find any differences in pathological tumor parameters when comparing optimists and pessimists. It appears that factors such as a positive surgical margin, pT-stage, or lymph node status do not influence patients’ perceptions of complications.

Marginally higher optimism scores for younger people have been reported before [13, 14]. Furthermore, males were slightly less optimistic than females in a German population survey of 9711 individuals from Hinz at al., [13]. In our study, there were no significant differences regarding age or gender distribution between optimists and pessimists. We also could not observe a significant difference regarding the rate of tertiary education between optimists and pessimists, whereas Hinz et al. reported lower optimism (LOT-R total) scores for people with lower levels of education [13]. Optimists had a significantly higher body mass index than pessimists. While pessimists were of normal weight in our study, optimists were overweight per definition [15].

A widely used instrument to measure optimism and pessimism in research is the Life Orientation Test-Revised (LOT-R), a 10-item scale that measures how optimistic or pessimistic people feel [16]. Plenty of positive effects on various aspects in life have been attributed to optimism. In a meta-analysis of 15 studies including 229,391 individuals, optimism was significantly associated with a decreased risk of cardiovascular events [17]. Optimism has also been associated with exceptional longevity (defined as survival to age 85 or older) in two epidemiologic cohorts of men and women [18]. In a study from Cohen et al., 334 healthy volunteers were given nasal drops containing rhinoviruses and monitored in quarantine for the development of a common cold. Increased positive emotional style was associated with a lower risk of developing a cold [19]. An optimistic attitude has also been associated with better health and quality of life in a Norwegian study of 1792 individuals aged 18–94 years [20]. Of note, our definitions of optimists and pessimists were not based on a validated test, but solely on the discrepancy between physician-assessed and patient-reported grading of complications.

### Role of the pre-operatively provided medical information

In our study, patients who subjectively reported higher complications than the physician-assessed grading (defined as pessimists) were significantly less likely to give the pre-operatively provided medical information a “very good” rating. Communication is a key element when handling complications, and a poor management can lead to complaints and litigation, as patients attempt to obtain the explanations that should have been provided for them initially [21]. In a study on patients’ and healthcare professionals’ perspectives on preoperative informed consent, the main identified barriers reported by patients were inadequate explanation about the intended procedure, family’s influence in the decision-making process, fear of surgery, fear of light or power interruption, inadequate time for discussion, and not letting family members attend the discussion [22]. The authors concluded that most of those barriers are avoidable and should be considered during preoperative informed consent [22]. Our results also suggest that the pre-operatively provided medical information may have a decisive influence on the patient´s subjective assessment of complications. Concepts like *shared decision-making* have been shown to decrease decisional conflict and increase trust in physicians [23] and might therefore also help patients to better comprehend postoperative complications.

### Limitations

Despite our thorough approach with prospectively collected data, this study has some limitations. Firstly, it was conducted in a single-center setting with a limited sample size. Secondly, the time delay until the interview could potentially influence patients’ subjective perceptions. We deliberately chose not to collect patients’ subjective views on complications immediately after discharge to avoid a recency effect. However, the three-month time lag could lead to recall bias in some patients. Furthermore, it would have been more accurate to collect patients’ perspective on preoperative medical information prior to surgery. Thirdly, a non-response bias may exist, as non-responders might include more dissatisfied patients. This is indicated by the higher CCI of dropped out patients (Supplementary Table 2). Moreover, there is no official definition of Clavien-Dindo ‘minor’ and ‘major’ complications. We have decided to grade Clavien-Dindo IIIa complications (intervention not under general anesthesia, such as a lymphocele puncture under local anesthesia) as minor, because it is significantly less invasive compared to a Clavien-Dindo IIIb complication (intervention under general anesthesia, such as an open revision for ileal anastomosis insufficiency). However, other definitions of ‘minor’ and ‘major’ complications might lead to different results. Additionally, how complications are communicated to patients could significantly influence their perceived severity. However, our study did not assess patient satisfaction with this aspect of communication.

## Conclusions

In our prospective study, two established complication grading systems (CDC and CCI) only correlated moderately with the patient´s subjective grading of complications. Half of the cystectomy patients had a realistic perception of the severity of postoperative complications. The number of optimists was three times higher than the number of pessimists. Our findings suggest that the preoperative medical information provided may have a decisive influence on the patient´s subjective assessment of complications. To the best of our knowledge, this is the first study that assesses the patient´s perspective on surgical complications in urological surgery. We suggest that the patient’s perspective should be included in the assessment of postoperative complications for a more comprehensive evaluation.

## Electronic supplementary material

Below is the link to the electronic supplementary material.

Supplementary Material 1

## Data Availability

No datasets were generated or analysed during the current study.

## Declarations

### Generative AI and AI-assisted technologies in the writing process

During the preparation of this work the authors used Microsoft Copilot in order to enhance the fluency of the text and to perform grammar checks. After using this tool/service, the author reviewed and edited the content as needed and take full responsibility for the content of the publication.
