# Supplementary material for: Discrepancies between physician-assessed and patient-reported complications after cystectomy – a prospective analysis
Source: World J Urol. 2025 Feb 10;43(1):115. doi: 10.1007/s00345-025-05487-7 (PMC11811455; doi:10.1007/s00345-025-05487-7)
Supplement: Supplementary file 1 — Supplementary Material 1 [file 345_2025_5487_MOESM1_ESM.docx]

**Supplementary data**

| **Q1** | How would you rate your in-hospital complications?   1. no complications 2. minor complications 3. major complications |
| --- | --- |
| **Q2** | How would you rate the preoperative medical information provided?   1. very good 2. good 3. satisfactory 4. sufficient 5. insufficient |

**Supplementary Table 1:** Questions of the standardized telephone interview after discharge to assess the patient’s perspective

| **Characteristic** | **In-analysis population,**  ***n* = 111** | **Drop-out population,**  ***n* = 43** | ***p*-value** |
| --- | --- | --- | --- |
| Age (years) | 71.3 (± 14.4) | 72.0 (± 12.0) | 0.41 |
| Gender |  |  |  |
| Male | 94 (85%) | 36 (83%) | 0.88 |
| Female | 17 (15%) | 7 (4.5%) |  |
| BMI (kg/m²) | 25.7 (± 5.5) | 26.3 (± 5.7) | 0.75 |
| ASA class >2 | 84 (76%) | 37 (86%) | 0.75 |
| Oncological indication | 99 (89%) | 41 (95%) | 0.23 |
| Neoadjuvant chemotherapy | 17/97 (18%) | 5 (12%) | 0.62 |
| Urinary diversion |  |  |  |
| Ileal conduit | 74 (67%) | 28 (65%) | 0.89 |
| Ileal neobladder | 36 (32%) | 15 (35%) |  |
| Ureterocutaneostomy | 1 (0.9%) | 0 (0%) |  |
| Organ-confined tumor (<T3a) | 65/97 (67%) | 22 (60%) | 0.46 |
| Positive lymph nodes | 17/89 (19%) | 9/31 (29%) | 0.25 |
| Positive surgical margin | 9/96 (9.4%) | 8/37 (22%) | 0.08 |
| Operation time (min) | 223 (± 97.5) | 204 (± 52) | 0.35 |
| Blood transfusion | 32 (29%) | 9 (21%) | 0.68 |
| IMCU/ICU nights | 1 (± 1) | 1 (± 1) | 0.26 |
| Hospital stay (days) | 18 (± 6.5) | 18 (± 5.5) | 0.20 |
| Educational level (ISCED) >4 | 50 (45%) | 11 (38%) | 0.54 |
| Clavien-Dindo Classification |  |  |  |
| None | 27 (24%) | 7 (16%) |  |
| Minor (I-IIIa) | 71 (64%) | 25 (58%) | 0.09 |
| Major (≥IIIb) | 13 (12%) | 11 (26%) |  |
| Comprehensive Complication Index | 20.9 (± 15.5) | 22.6 (± 32.3) | **0.03** |

**Supplementary Table 2:** Clinicopathological and sociodemographic characteristics of included patients vs. patients who dropped out. Values are presented as median ± interquartile range or n (%). Abbreviations: BMI: body mass index. ASA: American Society of Anesthesiologists. IMCU: intermediate care unit. ICU: intensive care unit. ISCED: International Standard Classification of Education. Neoadjuvant chemotherapy: patients with urothelial cancer considered
